# Laser Cut Interruption Detection from Small Images by Using Convolutional Neural Network

**DOI:** 10.3390/s21020655

**Published:** 2021-01-19

**Authors:** Benedikt Adelmann, Max Schleier, Ralf Hellmann

**Affiliations:** Applied Laser and Photonics Group, University of Applied Sciences Aschaffenburg, Wuerzburger Straße 45, 63739 Aschaffenburg, Germany; max.schleier@th-ab.de (M.S.); ralf.hellmann@th-ab.de (R.H.)

**Keywords:** laser cutting, remote sensing, convolutional neural network, cut interruption, image processing

## Abstract

In this publication, we use a small convolutional neural network to detect cut interruptions during laser cutting from single images of a high-speed camera. A camera takes images without additional illumination at a resolution of 32 × 64 pixels from cutting steel sheets of varying thicknesses with different laser parameter combinations and classifies them into cuts and cut interruptions. After a short learning period of five epochs on a certain sheet thickness, the images are classified with a low error rate of 0.05%. The use of color images reveals slight advantages with lower error rates over greyscale images, since, during cut interruptions, the image color changes towards blue. A training set on all sheet thicknesses in one network results in tests error rates below 0.1%. This low error rate and the short calculation time of 120 µs on a standard CPU makes the system industrially applicable.

## 1. Introduction

Cutting metals by fiber or disk lasers is nowadays a standard production process in the modern industry. While available laser powers rise continuously up to 30 kW or higher [1], cutting 100-mm-thick sheets is possible [2]. The most-often used laser powers are, however, 4 kW to 8 kW for cutting sheet thicknesses in the range from 0.3 mm to 10 mm with cut velocities between 10 mm/s to 1000 mm/s. Due to the general trend of higher automation, with the result of unmanned machines and the seamless combination of laser cutting machines with bending, separation or welding technologies, high and reliable quality of cuts are necessary to avoid downtime or damaging subsequent machine steps in such combined process chains.

The most usual quality defects influencing and hampering the subsequent machine steps are cut interruptions, burr formations and the high surface roughness of the cut edge, with interruptions being most objectionable. To obtain high-quality cuts, the process parameters, such as laser power, feed rate, gas pressure, working distance of the nozzle and focus position, respectively, must be selected appropriately. Imprecise process parameters and typical disturbance values like thermal lenses, unclean optics, damaged gas nozzles, gas pressure fluctuations and the variations of material properties may lead to poor-quality and, thus, nonconforming products. To ensure a high quality, an online quality monitoring system would be the best choice, which allows a quick response, reduces downtime or cost-extensive rework and saves material.

The usual monitoring methods are cameras or photodiodes to measure the optical, primary or secondary radiation from the cut kerf. In addition, some elaborate approaches like using a fiber Fabry-Pérot cavity microphone have been demonstrated [3]. To use such sensors as an industrial product, the sensor must be able to detect cut interruptions independent from the cutting direction, which is not feasible by below-bed sensors, as shown in [4,5]. For cut direction independent detection systems, in many publications, the sensor systems are integrated into the cutting head. Results by using photodiode-based sensors [6] showed that the mean photodiode’s current increases with lower cut qualities, while similar experiments [7] revealed increasing mean photodiode currents at lower cut surface roughness. Additionally, by using photodiodes [8], the burr height during laser cutting is calculated from the standard deviation of the photodiode current. From camera images of flame cutting with a CO_2_ laser, it is already possible to calculate the roughness, striation angle and the burr formation [3,9]. In detail, a NIR camera with a 40-Hz sampling rate was used, and the quality was calculated by the size of the hot process zone and the size of its circumscribed rectangle. The calculation time of 39 ms was quite high and not real-time applicable, especially at high feed rates.

A newer approach is to use a complex convolutional neural network to detect the burr formation during fiber laser cutting from images with 210 × 210 pixels [10], for which a burr detection accuracy of 92% has been reported. Such convolutional neural networks (CNN) are nowadays used very successfully for many image classification tasks, like face recognition and object detection [11,12] in medicine for cancer detection [13] or electroencephalogram (EEG) evaluations [14]; in geology for earthquake detection [15] and in many technical tasks, such as concrete crack detection [16], road crack detection [17], detecting wood veneer surface defects [18] or detecting wafer error determinations [19]. During laser welding, convolutional neural networks have been also successfully used to detect welding defects [20].

For cut interruption approaches, a polynomial logistic regression approach [21] is used to calculate the interruptions, based on laser machine parameters only. Photodiode-based methods for cut interruption detection are signal threshold-based [22], done by the comparison of different photodiodes [23] or based on cross-correlations [24]. However, all those methods have the disadvantage of requiring thresholds that vary with the sheet thickness or laser parameters or use a lot of subsequent samples that increase the reaction time. In addition, an adaptation to other materials or sheet thicknesses requires a large engineering effort. As a result, a learning system like cameras in combination with a CNN provides distinctive advantages. The learning images can be taken from cuts, which are necessary in order to determine the laser parameters for high-quality cutting anyway, so there is only little additional experimental effort. A camera system is also used in many laser machines to center the laser spot in the gas nozzle. Against this background, the target of this publication is to develop a reliable and fast CNN-based laser cut interruption detection that fulfills the industrial requirements. In particular, we designed a small neural network that classifies images of cuts and cut interruptions with a low error rate below 0.1% and a short calculation time of 120 µs.

## 2. Materials and Methods

### 2.1. Laser System

**Figure 1:**
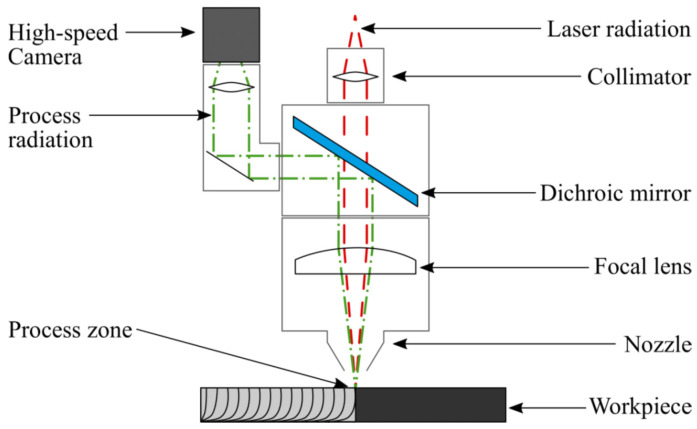
Optical setup of the cutting head.

The laser machine system is equipped with a 4-kW multimode fiber laser (IPG, Burbach, Germany) with a beam quality factor M² of 8.8, which determines the accessible focal diameter. In combination with a cutting head (Precitec, Gaggenau, Germany) with a 200-mm focus length, a focus spot diameter of 200 μm is achieved. Inside the cutting head, in between the collimation and the focus lens, a dichroic beam splitter reflects the visible thermal radiation from the process zone to the camera, as shown in Figure 1. Such optical setups with cameras are often included in laser cutting heads for adjusting the laser focus to exactly the middle of the gas nozzle. For the camera, no additional illumination is used in order save costs and weight for the light source and an additional beam splitter in order to increase the acceptability for industrial use.

To create videos of the cutting process, mild steel sheets with different thicknesses are laser cut by using nitrogen between 16 and 18 bar as the gas for the process. A summary of the used laser powers and feed rates (FR) is given in Table 1. For both the laser power and feed rate, five steps with a constant distance are used for each sheet thickness, resulting in a full factorial experimental design with 25 experiments (5 × 5). Typically, for laser cutting, a certain ratio between the laser power and feed rate is necessary for a successful cut (ratio for cut). This ratio increases with the sheet thickness. Below it, a cut interruption occurs, which is detected by about half of the overall performed experiments.

**Figure 2:**
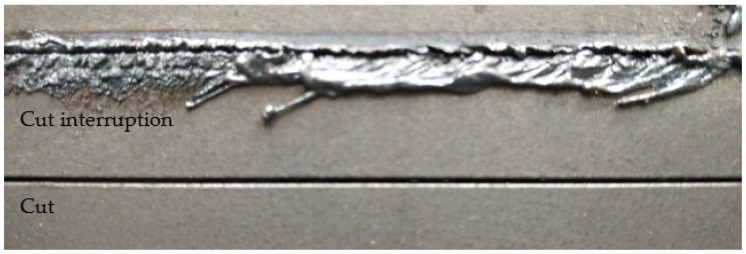
Top view of a metal sheet with a cut interruption (**top**) and complete cut (**bottom**).

During laser cutting, the laser melts the metal in a range determined by the focal area of the laser beam, and a gas jet blows the melt, once pierced, downwards out of the cut kerf so that the sheet is separated, as shown in the bottom part of Figure 2. When a cut interruption occurs, the bottom of the cut kerf is not hot enough to melt the metal completely. Therefore, the melt cannot be ejected downwards, partially stays in the kerf and welds the sheet together so that the sheets are not separated. The other part of the melt leaves the cut kerf on the top side and is deposited next to the kerf, as illustrated in the upper part of Figure 2. Therefore, cut interruptions can be easily identified by melt deposition on the top side.

### 2.2. Camera and Image Acquisition

For image acquisition, we used a high-speed camera (Fastcam AX50, Photron, Tokyo, Japan) with a maximum frame rate of 170,000 frames per second. The maximum resolution is 1024 × 1024 pixels, with a square pixel size of 20 × 20 μm^2^ in combination with a Bayer CFA Color Matrix. For process image acquisition, videos of the laser cut process are grabbed, with a frame rate of 20 kilo frames per second with an exposure time of 2 µs and a resolution of 256 × 256 pixels. Even at this high frame rate, no oversampling occurs, and consecutive images are not similar, because the relevant underlying melt flow dynamics are very fast and vary at estimated frequencies between 100 kHz and 300 kHz [25]. This estimation is further confirmed by the typical melt flow velocity, which is in the range of 10 m/s [26], resulting in our sampling rate between two images in a melt displacement of 0.5 mm, which is significantly higher than our spatial resolution given by the camera’s specifications.

**Figure 3:**
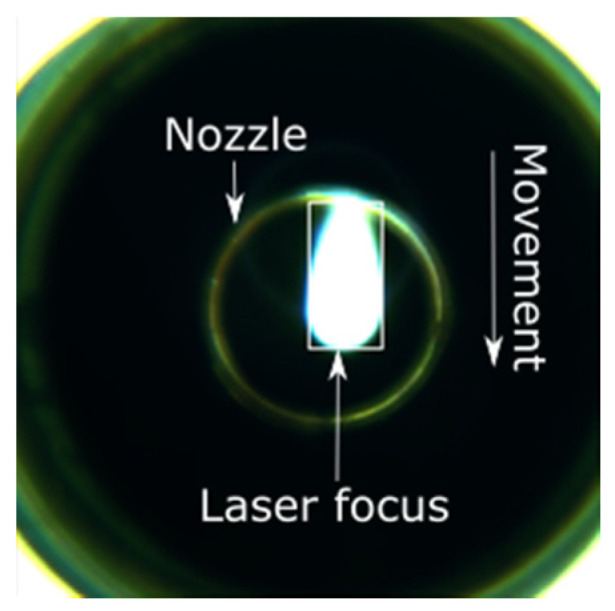
Original camera image.

Depending on the feed rate, each video consists of between 30,000 and 60,000 images. These videos also contain the acceleration and deceleration parts of the cut, i.e., the acceleration and deceleration phases of the linear drives of the laser cutting systems. As cut interruptions only occur during the phase of a high feed rate, the images of the acceleration and deceleration paths are removed from the videos, resulting in 8000 to 40,000 images per video. An example of such an image during a cut interruption is shown in Figure 3, revealing a large ring at the brinks that depicts the inner side of the cutting head, while the small ring in the middle reflects the nozzle. The top side of the cut kerf—and, therefore, the laser focus—is indicated by a bright area inside the nozzle. The movement direction in this image is downwards, so the tail behind the laser focus points upwards. To reduce the calculation time of the neural network from the image, a 32 × 64-pixel-sized image is extracted, which is indicated by the white rectangle in Figure 3. The size on the sheet of this rectangle is 640 μm in width and 1280 μm in length. The image width is in the range of the cut kerf of, typically, 500 to 600 μm. The direction of the cut kerf—and, therefore, the required rotation angle of the extracted image—can be taken from the actual movement direction of the machine drives. Due to the spectral behavior of the decoupling mirror, green is the dominating color.

Table 2 exemplarily shows two trimmed images of each sheet thickness during a cut and a cut interruption. It is obvious to see that, during a cut interruption, the image is brighter and the light area is larger as compared to the complete cuts. An evaluation of the brightness reveals for both cuts and interruptions a brightness fluctuation within one order of magnitude, even for two consecutive images. Furthermore, the brightness increases with the used laser power and the sheet thickness, so cut interruptions in 1-mm sheets look similar to cuts in 5-mm sheets, as depicted in Table 2. Attempts to classify images by brightness or the bright area resulted in a high error rate and were not recommendable. Methods using the calculations of a series of images result in lower error rates but increase the reaction time significantly. Therefore, convolutional neural networks are used to classify single images in order to achieve a low error rate and short reaction times.

To detect even short cut interruption with a length of 1 mm at maximum feed rates of 1000 mm/s, a camera with at least 1000 fps is necessary, which is not unusual for industrial cameras with a low resolution, as used here. Keeping in mind that cutting over a spike of the grid-based open mesh flooring, typically used in laser cutting machines, is similar to a cut interruption, a classification system requires a suppression of single-interruption signals. Typical floorings in laser machines have a mesh size between 5 cm to 10 cm. On this mesh, spikes are also placed in regular distances between 5 cm to 10 cm. With a typical spike size of 2 mm and a 5-cm mesh size in unfavorable cases, when cutting over a line of spikes, up to 4% (2 mm/5 cm) of the cut geometry may be located over the spikes. In the plane of the flat bed laser cutting machine, 0.16% (4%^2^) of the area is spikes. As a result, when cutting various arbitrary two-dimensional contours, up to 0.16% of the cuts may be located over a spike and can therefore be misinterpreted as cut interruptions. Considering this for a classification system, an error rate in the range of 0.1% or below is acceptable.

**Figure 4:**
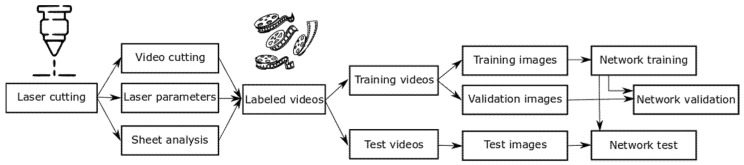
Experimental design of image provisions and evaluations.

The experimental design of our study is depicted in Figure 4, revealing the data flow. During laser cutting, the videos are recorded, and unnecessary data is removed, like frames after the end of the cut. The metal sheets are analyzed to detect whether a cut interruption occurred. This data is combined with the used laser parameters and the videos into a database of labeled videos. Therefore, each video has a corresponding pickle file; containing the experimental parameters of sheet thickness, laser power, federate, gas pressure and weather, the video depicts a cut or a cut interruption. From this database, different sets of videos with certain parameters—for example, sheet thickness or laser power—are taken for training and testing. The selection parameters are chosen in order to have videos of cuts and cut interruptions in both the training and test video sets. A percentage of the images taken from the training videos are evenly distributed and formed into an image vector and a label vector only containing whether this image shows a cut or a cut interruption. These image and label vectors are used for training, while, in the same way, another percentage of the images is taken from the training videos for validation. For testing the network, the image and label vectors are taken from the test videos to avoid similarity between the training data.

### 2.3. Hardware and Software

For learning and evaluating the neural network, a computer with an Intel Core 7-8700 with a 3.2-GHz clock rate in combination with a 16-GB DDR4 RAM was used. All calculations are performed with the CPU rather than the GPU to show that the machine learning steps are also possible to run on standard computers, which are usually integrated with laser cutting machines. The used software was TensorFlow version 2.0.0 in combination with Keras version 2.2.4.

### 2.4. Convolutional Neural Network Design

**Figure 5:**
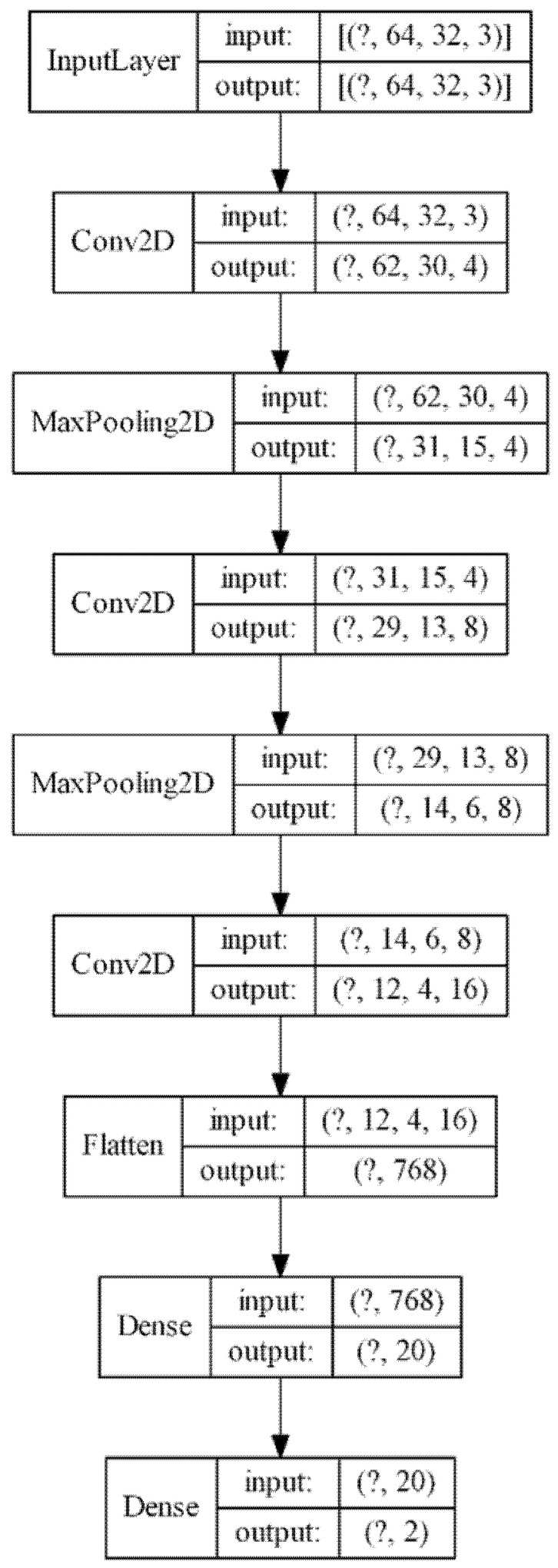
Design of the used convolutional neural network image.

The basic design of our CNN consisted of several alternating convolution and pooling layers, followed by fully connected layers [11,27], a design that has successfully been employed for other classification tasks of small images like the MNIST (Modified National Institute of Standards and Technology database) Dataset of handwritten digits [28]. To specifically adjust the network to our case, additional layers were added to improve the detection accuracy or removed to reduce the calculation time when they had no positive influence on the accuracy. The results of this optimization is shown in Figure 5, which is a small network similar to [20]. The model consists of three convolutional layers with a 3 × 3 kernel size and ReLU (Rectified Linear Unit) activation and two max pooling layers with a pool size of 2 × 2, also used by [20]. At the end of the network, a 20-node dense layer with ReLU activation and a 2-node dense layer with sigmoid activation were placed to produce class scores [27], which showed good performance in [29]. Details on how the layers work can be found in [27] . Changes in the design of our neural network result in a lower performance, like an additional fully connected layer at the end increases the calculation time by 10% without reducing the error rate, and one less convolutional layer increases the error rate by about 0.1 percentage points. Overall, our neural network consisted of 16,998 trainable parameters, with most of them (15,380) in the first dense layer.

The used model optimizer is Adam, which, according to [30] and together with the SDG (Stochastic Gradient Descent), provides superior optimization results. Furthermore, we used the loss function “sparse categorical cross entropy” to enable the categorical outputs and the metrics “accuracy”. With this network, the typical calculation time for training is between 220 µs and 260 µs per image and the epoch. For the evaluation, typically, 120 µs for each image is required. This is fast enough for laser cutting machines, which have a typical laser switch of times of 200 µs and several hundreds of milliseconds for drive deceleration. Even at high cutting velocities of 1000 mm/s, which is the maximum of typical laser cuts, the calculation time allows to calculate more than 1000 images per second, so small cut interruptions with a length in the range of 1 mm and below can also be detected.

## 3. Results

### 3.1. Required Training Effort

To avoid any overfitting and a lack of fit, exemplarily, a set of videos from cutting three-mm sheets at 3000-W laser power with 105,000 images overall was used for the training and validation of the neural network. From these images, 26% showed cut interruptions, while the others showed complete cuts. Out of these, 20% of the images of each video were used for training and the others for validation. Please note that, in order to also use the acceleration and deceleration paths of the laser cuts for training, the training images were evenly distributed over the videos, and the ratio between the cut interruptions and complete cuts remained unaltered. To test the network, images from other videos cutting at 2500-W laser power were used, with 48% of them showing cut interruptions while the others showed complete cuts. The calculation time for training, validation and testing together was about two minutes. The evaluation of the error rate as a function of the training epoch showed that the error rate for both the training and validation reached 0.001% after two epochs and remained there up to 100 epochs. The error rate during the tests on the 2500-W laser power images revealed 0.051% after two epochs and slightly increased to 0.071 after 100 epochs. These error rates were very low, even for a short training of two epochs. The error rate for the testing was significantly higher compared to the validation but still low enough for the application. The reason the error rate increased was the variation of the images by the lower laser power. In addition, the error rate for the testing increased slightly with the further learning steps, indicating an overfit on the training data at 2500 W, which reduced the accuracy at 3000 W. As a compromise between a lack of fit and overfit, for further training, a number of five epochs was used.

### 3.2. Necessary Number of Training Images

**Figure 6:**
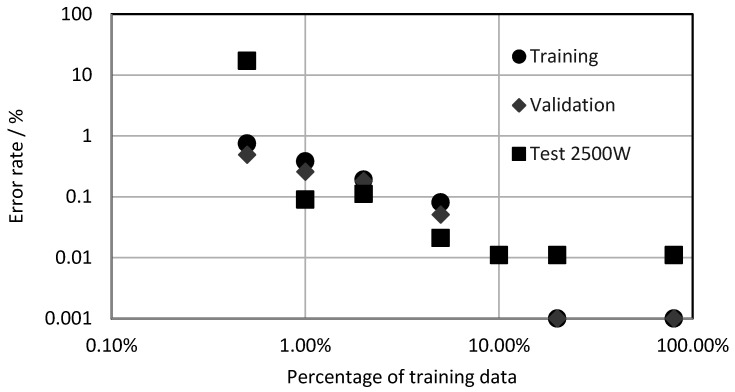
Error rate as a function of the training image percentage.

An important question is, how many images are necessary for a sufficiently low error rate? Therefore, the same videos as in the previous section were used with varying percentages of the training images. The resulting error rate as a function of the training image percentage is shown in Figure 6. Please note, in order to enable a logarithmic presentation, all evaluations with an error rate of zero were assigned to as 0.001%. As being typical for such training processes, all error rates decreased with the increasing training percentage [31]. Saturation occurred at about 10% to 20%, which represented 10–20-thousand training images. As a consequence, for all further trainings, at least 20-thousand images were used to ensure sufficient training data.

### 3.3. Comparison between Color Image and Greyscale

The camera system detects the thermal radiation during laser cutting, whose spectral emission characteristics are defined by Planck’s law. During laser cutting, the temperature fluctuates due to a wavelike melt flow and usually increases during a cut interruption. In addition, plasma plumes can occur, which can initiate a cut interruption or, often, a plasma formation itself, as a result of cut interruptions. Plasma plumes during laser cutting emit radiation, mainly in the ultraviolet-to-blue spectral region, with the latter, in fact, being detectable with the camera used in our setup. Thus, it can be assumed that a measurable blue shift indicates a cut interruption, which, in turn, can be utilized to improve the detection rate. Already, in Table 2, during cut interruptions, blue edges around the bright central image area can be observed. The disadvantage of color images compared to grayscale, however, is the higher calculation effort, memory demand and the accuracy is not necessarily higher [32].

**Figure 7:**
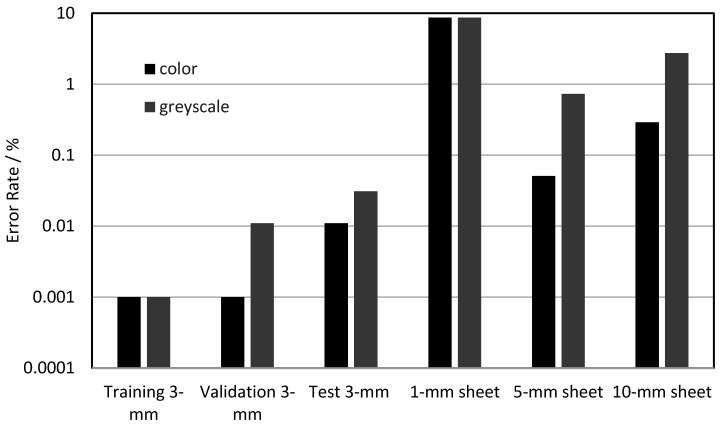
Error rate compared between the color images (here shown in black columns) and greyscale images (here shown as grey columns).

To determine a possible benefit of color images, the same training of cutting three-mm steel sheets using a laser power of 3000 W with 20% of the training images was performed on both color and grayscale images. The grayscale images were calculated from the color images with the OpenCV function “color_bgr2grey”. The training, validation (both at 3000 W, as in the previous sections) and test results at 2500 W are shown in Figure 7. Apparently, color images make only slight benefits to the error rate. In addition, in Figure 7 the model trained at a three-mm sheet thickness was tested with images from cutting other sheet thicknesses. As expected, the error rates for the other sheet thicknesses were significantly higher, which can be attributed to the apparently different images between the sheet thicknesses, as shown in Table 2. A remarkable result was the lower error rate for the color images at the transfer to thicker metal sheets. A possible reason for this effect is that color images have additional characteristics that are not so effective during validation because of the already low error rate. For the transfer to one-mm sheets, the results were, for both image types, very poor because of the low signal level and the smaller bright area. We attributed the missing advantage of the color images at the one-mm sheet thickness to the less distinctive blue shift for cut interruptions. Hence, due to the better transfer properties, for the continued investigations, color images were used, and a transfer of the learning results to other sheet thicknesses was accompanied by high error rates.

### 3.4. Dependence on Laser Power

**Figure 8:**
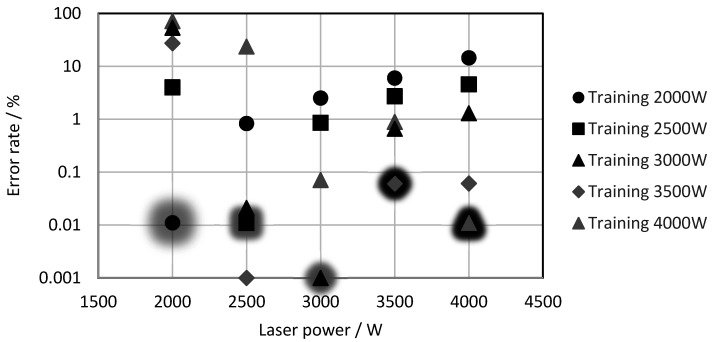
Error rate as a function of the laser power.

During laser cutting, usually, a constant laser power is applied. However, while cutting corners or low radii, the machine drives have to decelerate, which, in turn, requires a lower laser power to maintain the applied track energy and, thus, to avoid an inferior cutting quality. Therefore, it is necessary that the evaluation algorithm has a low error rate at various laser powers. The resulting question is, is it necessary to train for all used laser powers, or is training for one laser power enough for a certain sheet thickness? This is important, because producing learning videos for cuts and interruptions for several laser powers at each thickness is quite extensive. To answer this question, the neural network was trained for the three-mm sheets at different laser powers, and the test results at the various laser powers are shown in Figure 8. Please note that the tests at the trained laser powers are marked with shading. It was obvious that the error rates at the trained laser power and, often, at the adjacent laser powers were very low. With an increasing distance from the training set, the error rate rose sharply, up to levels of more than 10%. This means that, at different laser powers, the thermal radiation from the cut kerf and, therefore, the images changed significantly, so that the network was not able to classify the cuts and interruptions correctly.

To evaluate whether it is possible to correctly classify all the laser powers, the neural network was trained with 5% of the images of every video with cutting three-mm sheets. After training five epochs, a low training error rate of 0.05% and a validation error rate of 0.04% were achieved, which were only slightly higher as compared to the training and testing at the single laser power in Section 3.2. This means that one model for a certain sheet thickness can cover all different used laser powers that it was trained with at once. To test this model in a different way, a cut interruption was enforced by reducing the process gas pressure in four steps to 8 bar, where a cut interruption occurs. The resulting error rate of 0.02% revealed that the neural network was sufficiently generalized to also reliably detect cut interruptions for other reasons.

### 3.5. Generalization on All Sheet Thicknesses

In the previous section, we showed the possibility of successfully classifying images from different laser powers at a certain sheet thickness. In this section, we want to generalize further in order to classify all sheet thicknesses at all laser powers within the same network. Therefore, we trained the network on 2% of the images of every video from every sheet thickness and laser power, only omitting single videos for testing. This percentage was chosen to keep the number of learning images (82,000) in a similar range as compared to the previous sections, with the benefit of low training times. In contrast to the previous learning set, after five epochs, a 0.3% error rate still remained, which was much higher as compared to the training set for a single sheet thickness. The reason for this was the wide bandwidth of the images and the similarity of the cut interruptions of the low sheet thicknesses and cuts at the high sheet thicknesses. After further training, the error rate saturated at about 20 epochs, with a remaining error rate of 0.03%. A validation performed on the not-trained images from the same videos resulted in an error rate of 0.06%, which was a little higher compared to the validation for the network trained with one sheet thickness. A classification of the images from the videos omitted in the training phase revealed an error rate between zero and 0.09%. This error rates were very low, especially keeping in mind the similarity of the cut interruptions in the thin sheets and cuts in the thick sheets. Additionally, compared to burr detection during laser cutting with neural networks, which archived an error rate of 8% [10], our error rate was about two orders of magnitude lower. This highlights the good results of our cut interruption detection sets.

## 4. Discussion

The designed convolutional neural network was able to classify images from cuts and cut interruptions with a small error rate below 0.1% after short trainings of only two–five epochs. Additional epochs result in an overfitting that slightly increases the error rate of the tested images. A reason for the short training effort was the small size of the neural network, only 17-thousand parameters, which corresponded to a high ratio of the number of training images to the number of parameters in the neural network. To train the neural network with a low error rate, at least ten-thousand training images are necessary, which is in the same order of magnitude as the number of trainable parameters. The images differ strongly with the used laser power and sheet thickness, so the transfer of the training results to other sheet thicknesses or laser powers is limited but can be improved by the use of color images. As a consequence, the neural network has to be trained on every used sheet thickness and laser power, which requires a high experimental effort.

An analysis of the images revealed brighter images and larger bright areas during the cut interruptions, but the same effect can also occur when increasing the laser power or for cutting thicker sheets. This leads to similarities between cuts of thick sheets at high laser powers and cut interruptions of thin sheets at low laser powers. Despite this similarity, the neural network was able to classify cuts and cut interruptions with a low error rate below 0.1% over a dataset with varying sheet thicknesses and laser powers. Further studies shall address the transfer and application of our sensor and CNN approach to other materials, such as, e.g., aluminum or brass, and, also, take into account machine conditions like polluted, unclean focusing optics.

A successful detection scheme for cut interruptions, as presented here, improves the resource efficiency of laser-based production technologies by reducing the material loss and objectionable rework by miscuts, avoids downtime or the damaging of subsequent machine steps in such combined process chains and improves the energy efficiency during the cutting process by setting proper parameters, respectively.

## 5. Conclusions

In this publication, we used a small convolutional neural network with a calculation time of 120 µs to classify images from the processing zone during laser cutting into complete cuts or cut interruptions. The images with a size of 32 × 64 pixels were taken from more than 100 videos of laser cutting steel sheets with different thicknesses, laser powers and feed rates. We found that five epochs of training were a good compromise between enough training and a low overfit and resulted in a small error rate of 0.05% for training a single sheet thickness at a certain laser power. The transfer of this result to other laser powers was limited to small power differences, so training on several laser powers was necessary, which resulted in error rates lower than 0.05%. A comparison between the color images and greyscale images revealed slight advantages for the color images at the same sheet thickness and larger advantages when transferring to other sheet thicknesses; thus, color images are preferable. Despite the similarity of cuts in thick sheets and cut interruptions in thin sheets, training on all sheet thicknesses and laser powers in one network also resulted in a low error rate below 0.1%, which is industrially applicable.

## Figures and Tables

**Table 1:** Parameter spaces for laser cutting. FR: feed rate.

Thickness	Min. Power	Max. Power	Power Step	Min. FR	Max. FR	FR Step	Ratio for Cut
1 mm	500 W	2500 W	500 W	100 mm/s	200 mm/s	25 mm/s	10 W/mm/s
3 mm	2000 W	4000 W	500 W	50 mm/s	90 mm/s	10 mm/s	40 W/mm/s
5 mm	2000 W	4000 W	500 W	20 mm/s	40 mm/s	5 mm/s	100 W/mm/s
10 mm	3000 W	4000 W	250 W	11 mm/s	19 mm/s	2 mm/s	210 W/mm/s

**Table 2:** Cut images for different sheet thicknesses and cut states.

	1 mm	3 mm	5 mm	10 mm
Cut	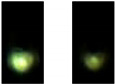	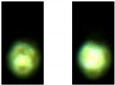	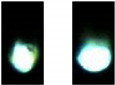	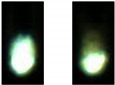
Interruption	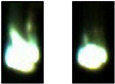	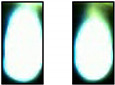	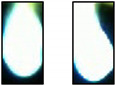	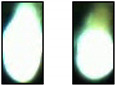

## Data Availability

The data presented in this study are available on request from the corresponding author. The data are not publicly available due to the high data size.

## References

[1] Tamura K., Ishigami R., Yamagishi R. (2015). Laser cutting of thick steel plates and simulated steel components using a 30 kW fiber laser. J. Nucl. Sci. Technol..

[2] Shin J.S., Oh S.Y., Park H., Chung C.-M., Seon S., Kim T.-S., Lee L., Lee J. (2018). Laser cutting of steel plates up to 100 mm in thickness with a 6-kW fiber laser for application to dismantling of nuclear facilities. Opt. Lasers Eng..

[3] Wen P., Zhang Y., Chen W. (2012). Quality detection and control during laser cutting progress with coaxial visual monitoring. J. Laser Appl..

[4] Alippi C., Bono V., Piuri V., Scotti F. (2002). Toward real-time quality analysis measurement of metal laser cutting. 2002 IEEE International Symposium on Virtual and Intelligent Measurement Systems (IEEE Cat. No.02EX545).

[5] Garcia S.M., Ramos J., Arrizubieta J.I., Figueras J. (2020). Analysis of Photodiode Monitoring in Laser Cutting. Appl. Sci..

[6] Levichev N., Rodrigues G.C., Duflou J.R. (2020). Real-time monitoring of fiber laser cutting of thick plates by means of photodiodes. Procedia CIRP.

[7] Schleier M., Adelmann B., Neumeier B., Hellmann R. (2017). Burr formation detector for fiber laser cutting based on a photodiode sensor system. Opt. Laser Technol..

[8] Sichani E.F., de Keuster J., Kruth J., Duflou J. (2012). Real-time monitoring, control and optimization of CO_2_ laser cutting of mild steel plates. Proc. Int. Matador Conf..

[9] Sichani E.F., de Keuster J., Kruth J.-P., Duflou J.R. (2010). Monitoring and adaptive control of CO_2_ laser flame cutting. Phys. Procedia.

[10] Franceschetti L., Pacher M., Tanelli M., Strada S.C., Previtali B., Savaresi S.M. Dross attachment estimation in the laser-cutting process via Convolutional Neural Networks (CNN). Proceedings of the 2020 28th Mediterranean Conference on Control and Automation (MED).

[11] Guo Y., Liu Y., Oerlemans A., Lao S., Wu S., Lew M.S. (2016). Deep learning for visual understanding: A review. Neurocomputing.

[12] Voulodimos A., Doulamis N., Doulamis A., Protopapadakis E. (2018). Deep Learning for Computer Vision: A Brief Review. Comput. Intell. Neurosci..

[13] Ting F.F., Tan Y.J., Sim K.S. (2019). Convolutional neural network improvement for breast cancer classification. Expert Syst. Appl..

[14] Acharya U.R., Oh S.L., Hagiwara Y., Tan J.H., Adeli H. (2018). Deep convolutional neural network for the automated detection and diagnosis of seizure using EEG signals. Comput. Biol. Med..

[15] Perol T., Gharbi M., Denolle M. (2018). Convolutional neural network for earthquake detection and location. Sci. Adv..

[16] Dung C.V., Anh L.D. (2019). Autonomous concrete crack detection using deep fully convolutional neural network. Autom. Constr..

[17] Zhang L., Yang F., Zhang Y.D., Zhu Y.J. Road crack detection using deep convolutional neural network. Proceedings of the 2016 IEEE International Conference on Image Processing (ICIP).

[18] Urbonas A., Raudonis V., Maskeliūnas R., Damaševičius R. (2019). Automated Identification of Wood Veneer Surface Defects Using Faster Region-Based Convolutional Neural Network with Data Augmentation and Transfer Learning. Appl. Sci..

[19] Nakazawa T., Kulkarni D.V. (2018). Wafer Map Defect Pattern Classification and Image Retrieval Using Convolutional Neural Network. IEEE Trans. Semicond. Manuf..

[20] Khumaidi A., Yuniarno E.M., Purnomo M.H. Welding defect classification based on convolution neural network (CNN) and Gaussian kernel. Proceedings of the 2017 International Seminar on Intelligent Technology and Its Applications (ISITIA).

[21] Tatzel L., León F.P. (2019). Prediction of Cutting Interruptions for Laser Cutting Using Logistic Regression. Lasers Manuf. Conf..

[22] Adelmann B., Schleier M., Neumeier B., Hellmann R. (2016). Photodiode-based cutting interruption sensor for near-infrared lasers. Appl. Opt..

[23] Adelmann B., Neumeier B., Schleier M., Wilmann E., Hellmann R. Optical cutting tear detection system for industrial fiber laser based cutting machines. Proceedings of the Lasers in Manufacturing 2015 Proceedings.

[24] Schleier M., Adelmann B., Esen C., Hellmann R. (2018). Cross-Correlation-Based Algorithm for Monitoring Laser Cutting with High-Power Fiber Lasers. IEEE Sensors J..

[25] Tennera F., Klämpfla F., Schmidta M. How fast is fast enough in the monitoring and control of laser welding?. Proceedings of the Lasers in Manufacturing Conference.

[26] Arntz D., Petring D., Stoyanov S., Quiring N., Poprawe R. (2018). Quantitative study of melt flow dynamics inside laser cutting kerfs by in-situ high-speed video-diagnostics. Procedia CIRP.

[27] O’Shea K., Nash R. An Introduction to Convolutional Neural Networks. http://arxiv.org/pdf/1511.08458v2.

[28] An S., Lee M., Park S., Yang H., So J. (2008). An Ensemble of Simple Convolutional Neural Network Models for MNIST Digit Recognition. arXiv.

[29] Acharya U.R., Oh S.L., Hagiwara Y., Tan J.H., Adam M., Gertych A., Tan R.S. (2017). A deep convolutional neural network model to classify heartbeats. Comput. Biol. Med..

[30] Bello I., Zoph B., Vasudevan V., Le Q.V. Neural Optimizer Search with Reinforcement Learning. http://arxiv.org/pdf/1709.07417v2.

[31] Keshari R., Vatsa M., Singh R., Noore A. Learning Structure and Strength of CNN Filters for Small Sample Size Training. Proceedings of the IEEE Conference on Computer Vision and Pattern Recognition (CVPR).

[32] Sachin R., Sowmya V., Govind D., Soman K.P., Thampi S.M., Krishnan S., Corchado Rodriguez J.M., Das S., Wozniak M., Al-Jumeily D. (2018). Dependency of Various Color and Intensity Planes on CNN Based Image Classification. Advances in Intelligent Systems and Computing, Advances in Signal Processing and Intelligent Recognition Systems.

